# Deducing Protein Function by Forensic Integrative Cell Biology

**DOI:** 10.1371/journal.pbio.1001742

**Published:** 2013-12-17

**Authors:** William C. Earnshaw

**Affiliations:** Wellcome Trust Centre for Cell Biology, University of Edinburgh, ICB, Edinburgh, Scotland, United Kingdom

## Abstract

Eminent cell biologist Bill Earnshaw visualizes a future in which forensic cell biologists will prowl the ruins of abandoned high-throughput datasets, extracting key data to create classifiers they will use to predict the functions of both known and previously uncharacterized proteins.

**Summary**

Our ability to sequence genomes has provided us with near-complete lists of the proteins that compose cells, tissues, and organisms, but this is only the beginning of the process to discover the functions of cellular components. In the future, it’s going to be crucial to develop computational analyses that can predict the biological functions of uncharacterised proteins. At the same time, we must not forget those fundamental experimental skills needed to confirm the predictions or send the analysts back to the drawing board to devise new ones.

What does it mean for cell biologists to be working in a “post-genomic” era? This is more a media term than a scientific term, but I believe that what is commonly understood by it is that we now work in an era where there is claimed to be a complete—or near complete—parts list for the cells of most major experimental organisms. Of course this belief is not entirely true. We all know that the genome projects have not given us the complete sequence of all human (or any other metazoan) DNA. For example, we still do not have a contiguous sequence of the highly repetitive regions of chromosomes in and around centromeres and at a number of other loci. Of course, there may not be very many (or even any) important undiscovered protein-coding genes hiding in these regions, but it is likely that there are still a substantial number of unknown proteins encoded by the human genome. Furthermore, as I will discuss below, there are also likely to be quite a few proteins whose functions we think we know, but that have important or even essential alternative functions that are currently unsuspected.

The subject of this essay is an important emerging area in cell biology research: how to predict the functions of uncharacterised and unknown proteins and how to identify and characterise novel functions of known proteins (for earlier discussions of this see [1]–[3]). These are areas that I predict will involve the coordination of very different kinds of advances by two distinct cohorts of future cell biologists. The first of these will be adept at producing a huge range of information from a wide variety of “omics” and other high-throughput studies and able to integrate this information to predict how proteins function. The second will devise low and high-throughput biochemical tests to prove or disprove those predictions in the laboratory.

Before I go further, I should define my terms. First, the term “function” means different things to different groups of researchers: to a classical geneticist, for example, it might mean turning a fly’s antennae into legs; to a biochemist it might mean forming a complex with a group of other proteins known to be involved in a particular process such as regulation of gene expression, and to a structural chemist it might mean removing an electron from one chemical bond and transferring it to another. As a cell biologist, I am usually content that I know something about the function of my protein if I know where it is in the cell, what other proteins it interacts with, and what part it plays in the particular cellular process I happen to be studying. Depending on the organism, the functions of some 20%–60% of proteins are uncertain [2].

As referred to here, “uncharacterised proteins” are proteins that are present in annotated databases, but whose functions are not determined. Proteins published as, for example, “protein up-regulated in cancer” or “protein up-regulated in cell type x” may have names, but no one actually knows what they do. In a recent study of the proteome of mitotic chromosomes, amongst >4,000 proteins identified, my colleagues and I found just over 300 proteins like this [4].

What I call “unknown proteins” can be of two classes. First, many proteins are present in databases but have not yet appeared in publications or been given formal names. In our chromosome analysis, we identified 260 of these unknown proteins. The second class comprises proteins whose existence is unsuspected, that is, of course, until someone describes them. For example, by using mass spectroscopy Crispin Miller’s group found 346 novel peptides and proteins that were smaller than the minimum cut-off size used for identifying protein-coding genes by the Human Genome Project [5]. Shortly thereafter, the same group identified 39 previously unsuspected genes encoding novel short proteins in *Schizosaccharomyces pombe* [6]). Alternative splicing, where a single pre-mRNA can yield several—or many—functional mature mRNAs that can encode a range of related proteins, some of which may have very different functions, provides another very rich source of previously unknown proteins.

RNA-seq and proteomics studies are just now beginning to reveal the true complexity of proteins that can be generated from the ∼20,000 protein-coding genes in humans. Beyond the discovery of unknown proteins, there is a world of largely unexplored functional diversity arising from post-translational modifications of proteins. Given the example that simple phosphorylation can change the nuclear lamins from members of a highly insoluble structural framework underpinning the nuclear envelope to soluble proteins in the mitotic cytoplasm [7],[8] and the vast numbers of modifications on proteins such as the histones [9], the scope for functional complexity induced by post-translational modifications is truly vast and will keep cell biologists busy for many years to come.

Exploring the unknown is always exciting and challenging, but occasionally exploring the “known” can yield equally exciting dividends. In some cases it is straightforward to predict proteins that will have multiple functions—an obvious example lies in the correctly, but perhaps naively named histone acetyl transferases and histone deacetylases and their ilk, which likely add and remove post-translational modifications to very large numbers of cellular proteins in addition to histones. Other examples of proteins that “moonlight” with a second function are not so easy to guess in advance. One example is cytochrome c, whose place in the world as a member of the electron transport chain was comfortably established and thought to be fully understood when I first studied undergraduate biochemistry in the 1970s—that was until Xiaodong Wang and his team discovered that it also has an essential role in assembly of the apoptosome during apoptotic cell death [10]. Cytochrome c is not alone in moonlighting. Many of the lens crystallins, for example, are related to known enzymes that function in metabolic pathways and stress responses [11]. How many other proteins, whose functions have been determined with much less precision than that of cytochrome c, actually have other essential functions in cellular processes? At present we have no way of telling; however, I suspect and predict that the number is not small. It is not at all unreasonable to imagine that one reason why humans have fewer genes than one might guess is that quite a few proteins do more than one job. How will we ever figure this out other than by chance observation or coincidence?

## The Power of Making Lists

By far the simplest way to predict the function of an unknown protein is to show that it has significant sequence relatedness to another protein whose function is known. This is all well and good unless your favourite protein is a “pioneer” (i.e., protein of unknown function whose amino acid sequence is unrelated to any protein of known function), in which case you are on your own without a tried and tested recipe for how to proceed. I suggest that in the future the emerging art of predicting the function of unknown proteins (and predicting novel functions for known proteins) will be the domain of “forensic integrative cell biologists” (using “forensic” as defined in Wikipedia as the investigation of “situations after the fact, and to establish what occurred based on collected evidence”). These researchers will develop methods for integrating a wide range of approaches to look at protein function, breaking each approach down to its component “classifiers”: lists of all the components of a system (or subsystem, as defined by [3]) identified by a particular experiment, each of which is attributed a score based on a predefined criterion and then ranked in order of that score. The key is then to figure out how to combine and compare these classifiers to identify patterns that can be used to deduce functional relationships amongst groups of known proteins and use those patterns to characterise the behaviour of specific uncharacterised proteins.

This process of deducing protein function through “guilt by association” has worked spectacularly well in identifying novel cell cycle genes. One such analysis involved large numbers of cDNA libraries from a wide range of different cell types and under different growth conditions, characterising the distribution of observed cDNAs for known cell cycle genes across those libraries, and then looking for cDNAs encoding proteins of unknown function that exhibited a similar distribution across the many datasets [12]. This analysis identified eight proteins likely to function in the cell cycle. Among them, CDCA1 was later identified as Nuf2, a member of the NDC80 complex responsible for attaching microtubules to kinetochores [13],[14]; CDCA2 was later identified as Repo-Man, an important targeting subunit of protein phosphatase 1 [15],[16]; CDCA5, was later identified as sororin, an important regulator of sister chromatid cohesion [17]; and CDCA8 was later identified as Borealin/Dasra, an essential member of the chromosomal passenger complex [18],[19].

**Figure 1:**
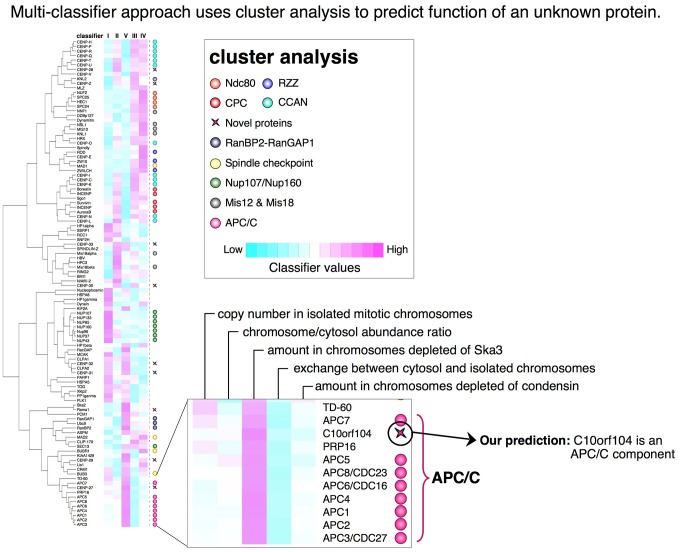
Use of a multi-classifier approach to predict the function of a novel protein. C10orf104 was a novel protein associated with mitotic chromosomes whose amino acid sequence offered no clues to its functions. When its association with isolated mitotic chromosomes was investigated using five different types of proteomic experiments, the protein was shown to cluster with APC/C components [4]. This led us to predict that C10orf104 might be a novel APC/C component—a prediction that was confirmed by studies carried out independently in three other groups [24]–[26]. The protein is now known as APC16.

The development of multi-classifier combinatorial proteomics for the analysis of the mitotic chromosome proteome [4] is an example of applying this type of approach to other sorts of datasets. In this case, we combined data (arrayed as classifiers) from five different types of proteomics experiments, all based on the stable incorporation of labelled amino acids in culture (SILAC) approach [20], to look at the association of several thousand proteins with isolated mitotic chromosomes. These classifiers included the estimated number of copies of each protein in mitotic chromosomes; the ratio of the amount of each protein in isolated chromosomes versus the amount in the cytosol after removal of the chromosomes; the extent to which proteins present in cytosol bound to isolated chromosomes that had been incubated in crude cytosol; the extent to which the abundance of particular proteins on chromosomes was affected by loss of the condensin complex from chromosomes; and the extent to which the abundance of particular proteins on chromosomes was affected by loss of the SKA complex from chromosomes [4]. We constructed classifiers in which the proteins from these different experiments were ranked according to their SILAC ratios (e.g., whether there was more or less of a given protein associated with chromosomes in a particular experiment) and subjected those classifiers to cluster analysis, which has been widely used to analyse patterns in microarray experiments [21] and has since been put to many other uses [4],[22],[23]. In that cluster analysis, we consistently observed that members of functionally related protein complexes tend to cluster together. In particular, a protein that was uncharacterized at that time, called C10orf104, clustered amongst the subunits of the anaphase promoting complex (APC/C), leading us to speculate that this protein was a component of that complex (Figure 1). This speculation was subsequently confirmed when C10orf104 was identified by others as a novel component of the APC/C–APC16 [24]–[26].

Our cluster analysis initially used proteomics data based solely on protein abundance in mitotic chromosomes. In future functional analyses, however, there need be no requirement to limit the inputs to one type of data. Any dataset where proteins can be assigned a numerical score based on some sort of systematic measurement can be used to construct a classifier. For example, bioinformatic analysis of the amino acid sequence can be used to devise a classifier to score large numbers of proteins. In our study of mitotic chromosomes, we scanned the sequences of all proteins recognised in mitotic chromosomes for documented amino acid sequence motifs. We then scored those motifs for their distribution between known cytoplasmic proteins and known chromosomal proteins. This enabled us to rank each motif depending on how associated it was with chromosomal proteins versus cytoplasmic proteins. This ranked list for all of the proteins in the dataset was then used as a classifier, scoring proteins on how “chromosome-like” their sequence motifs were [4]. This sort of analysis potentially expands the utility of amino acid sequence information beyond the obvious (and sometimes not so obvious) comparisons of sequences between different proteins.

**Figure 2:**
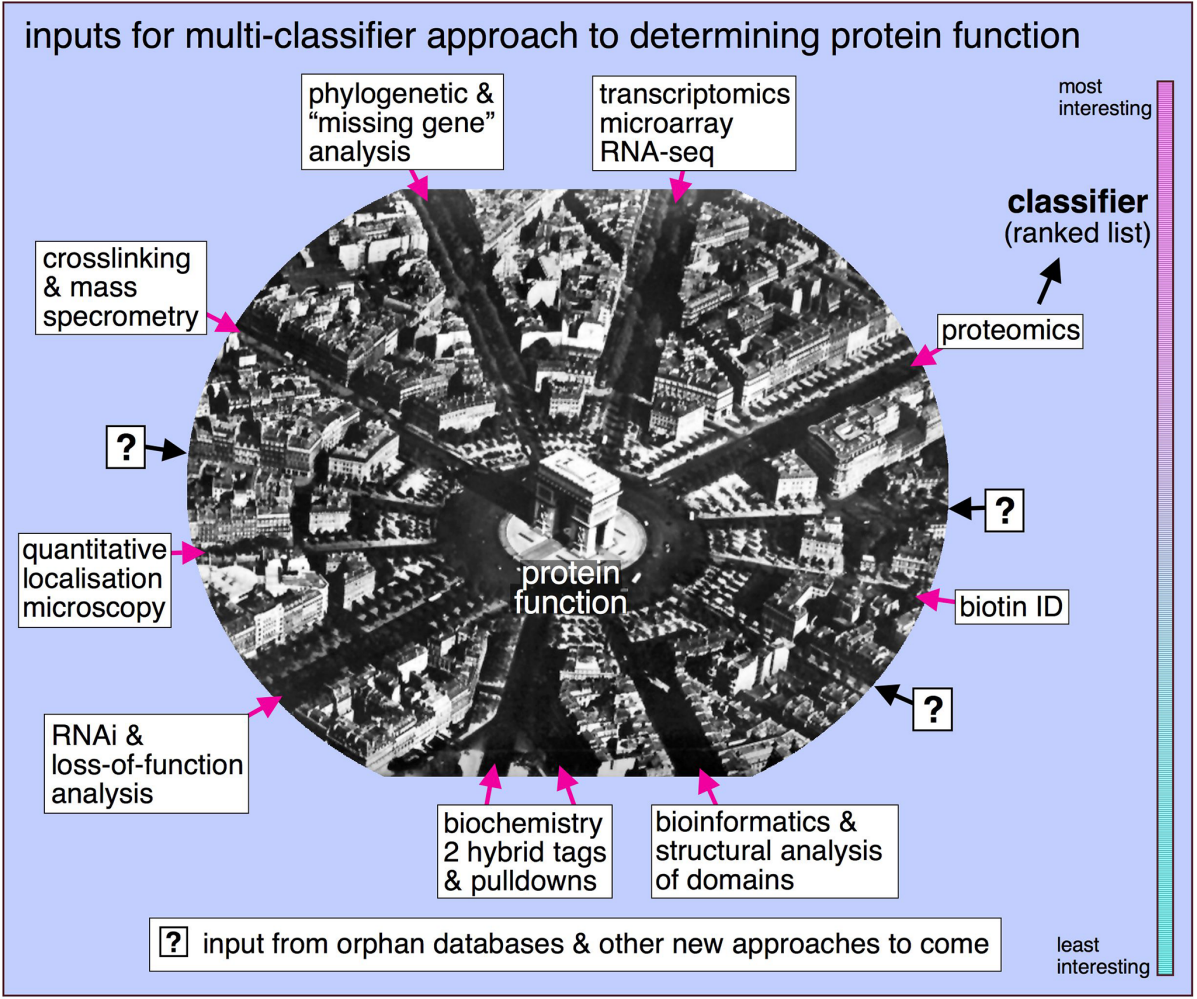
A multi-classifier approach to determining protein function. In this emerging approach to determine function, a variety of very different experimental approaches are used to make lists, called classifiers, in which proteins are given numerical scores according to the parameters being examined and ranked in numerical order. These ranked lists may then be combined by using clustering analysis, as in Figure 1, or analysed by other sorts of machine learning algorithms to look for common patterns that provide clues to functional relationships.

Beyond analysis of sequence motifs, other types of data that could be combined to make classifiers include: comparative evolutionary (often called genome context) data (including homologous and orthologous proteins and synteny relationships—see [1],[2]); phenotypes in genome-wide RNAi screens (for two examples, see [23],[27]); expression patterns in different cells and tissues or in response to various stimuli or in different phases of the cell cycle (for one of many possible examples, see [28]); localisation within cells and protein interaction data, including two-hybrid screens and pulldown experiments (see [25]). This is by no means a comprehensive list. Any way of analysing a large cohort of proteins that gives rise to a quantitative comparative ranking can provide the basis for a novel classifier (Figure 2).

Where significant conceptual breakthroughs will be required in the future will be in deciding how to combine these classifiers to best elucidate functional relationships between proteins. Our original efforts simply hijacked an algorithm for cluster analysis that was originally developed for comparing microarray data. We subsequently exploited the power of machine learning including “random forest” analysis to look for wider relationships between groups of proteins [4]. (Random forest analysis is a sort of machine learning based on the construction and use of random decision trees to examine relationships between data. For a helpful description, see http://en.wikipedia.org/wiki/Random_forest.) In the future, novel algorithms and approaches should yield more powerful ways to reduce these multidimensional datasets to specific and testable predictions of function—a kind of multidimensional guilt-by-association analysis.

## Excavating Abandoned Datasets

If all this explains why I believe the future will belong to integrative cell biologists, why did I say “forensic” integrative cell biologists? This additional term addresses the amazingly wasteful approach that the scientific community currently takes to high-throughput analyses. A typical high-throughput analysis—let’s take the example of a proteomics analysis—will conduct a large number of experiments generating data on literally thousands of proteins. To publish this analysis, the researchers typically focus on some specific readout from the experiment—for example, identification of one or several novel proteins whose localisation or function is of interest. They then write a paper describing the screen, after going on to “work up” only one or a few of their novel hits, the latter providing the functional detail demanded by most top journals.

All of this is fine, but what comes next? The authors may, if they are both talented and lucky, publish their paper in a high-impact journal, thereby accruing valuable “job points” for the first and senior authors. The massive dataset is available on line, of course, typically in a huge table that can be accessed by the well informed, if they have sufficient motivation. But I will bet (and this is definitely a statement made with no data to back it up), that those huge tables are accessed very seldom and in many instances not at all: not by other competing labs and probably not by the original lab either. One reason for this may be the perception that it is quite unlikely that a second or third high-impact paper is going to come from the original dataset. After all, if that screen has been already been described and published, what novelty can remain in there?

It seems to me that one consequence of the recent fashion for high-throughput analyses by proteomics, transcriptomics, RNAi, localisation—you name it—is the production of large numbers of orphan datasets that are accessible, but sit abandoned, poorly curated and ignored on servers around the scientific world. I maintain that these datasets constitute a truly vast untapped resource that is waiting to be plundered by forensic integrative cell biologists to generate huge numbers of powerful classifiers that can add to our knowledge of functional relationships between proteins. I believe that we need to move from the present situation of single-use disposable datasets to a future of multi-use data. “Same data, different question” should be a watchword of future forensic integrative cell biologists. Admirable efforts have been made in this direction for some model organisms, notably, *Drosophila* (FlyBase, http://flybase.org/), budding yeast (*Saccharomyces* Genome Database, http://www.yeastgenome.org/), *Caenorhabditis elegans* (Wormbase, http://www.wormbase.org/#01-23-6), and zebrafish (ZFIN: The Zebrafish Model Organism Database, http://zfin.org/). There are also servers that attempt to link multiple datasets, including GO (http://www.geneontology.org/), DAVID (http://david.abcc.ncifcrf.gov/), String (http://string-db.org/), and Stitch (http://stitch.embl.de/); but although these are extremely valuable and constantly being improved, they are far from comprehensive.

I believe that this problem needs to be developed as a priority by funding agencies who are currently investing large sums in open-access publishing but not (as far as I know) into constructing a systematic framework in which all high-throughput data can be made widely available in useful form. If it is wasteful for grant-holders to pay to access articles whose contents were funded by the same granting agencies, then it is surely equally—if not more—wasteful for those agencies to fund expensive studies whose major data output is effectively mothballed after a single use.

## My Kingdom for a Biochemist

The most brilliant integrative computational analysis can produce a potentially paradigm-altering hypothesis (otherwise known as a guess), but what it cannot do is design, execute, and interpret the experiments that are going to prove or disprove that hypothesis. Despite all the wonderful high-tech, massively parallel analyses that are presently employing the robots of the biological world, there will always be a place for that humble workhorse, the biochemist.

The scientists of the future who will validate the predictions of protein function generated by multidimensional multi-classifier analyses will be biochemists and cell biologists who are trained to sink their teeth into a protein and not let go until they have demonstrated its function *in vitro*, in cells, and *in vivo*. It does no good to predict function if we cannot test our guess. Thus, biochemistry, which is regarded in some quarters as old-fashioned, must remain strong in coming years as a critical area of endeavour that will confirm some predictions made by the integrative cell biologists while refuting others. In my opinion, biochemistry is not a hobby, like cloning, that one does on the side. To express, purify, and analyse proteins and protein complexes is an art that requires dedication, training, and practice. The training is rigorous and not for the timid, but the outcome is well worth the effort as mechanisms predicted by classifier analyses of “omics” and high-throughput datasets are tested and the workings of cellular machines elucidated.

## Abbreviations

APC/C: anaphase promoting complex/cyclosome
